# Supplementary figures and images for: Biomarker Signatures of Quality for Engineering Nasal Chondrocyte-Derived Cartilage
Source: Front Bioeng Biotechnol. 2020 Apr 7;8:283. doi: 10.3389/fbioe.2020.00283 (PMC7154140; doi:10.3389/fbioe.2020.00283)

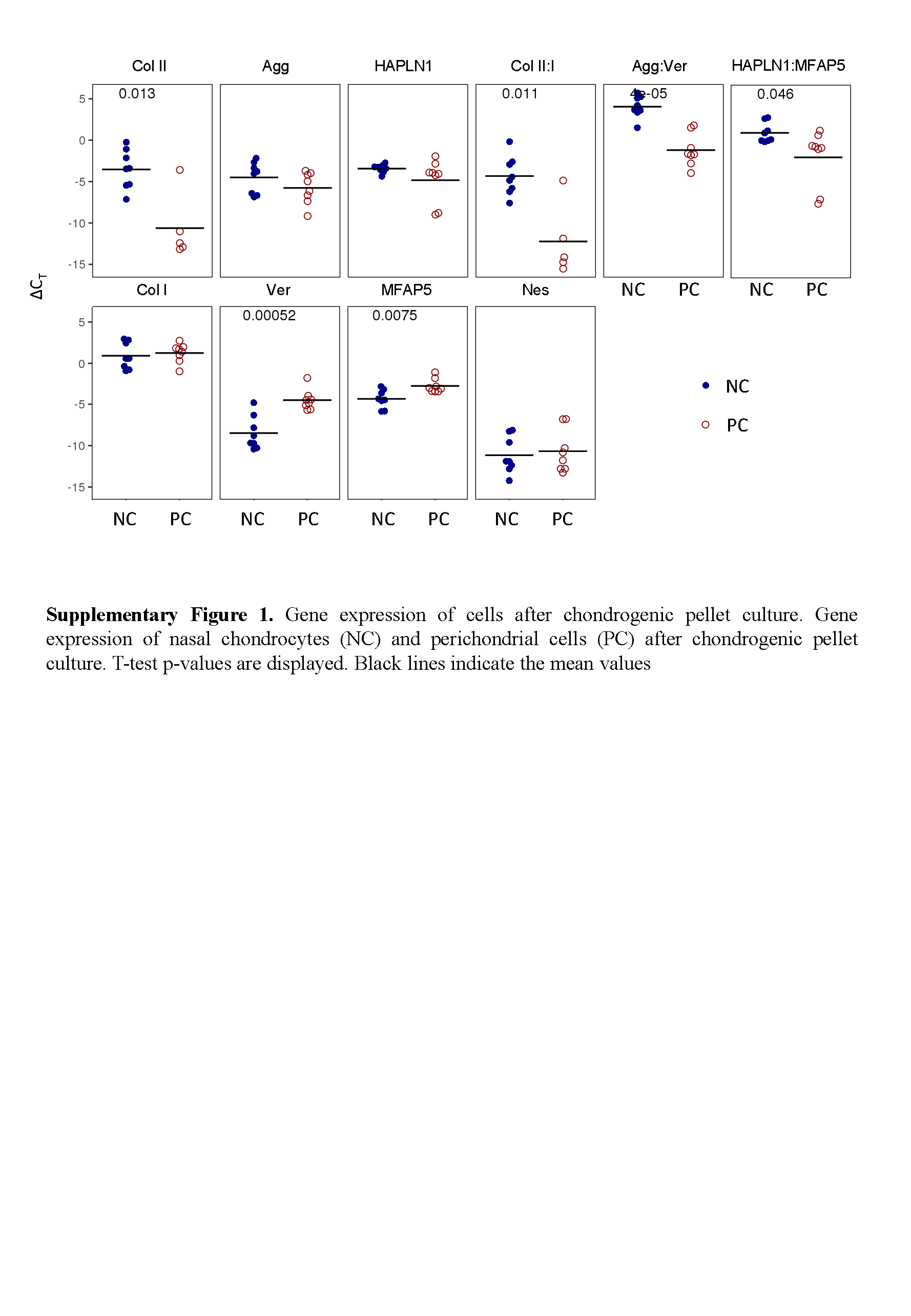

Supplement: Supplementary file 1 [file Image_1.TIFF]

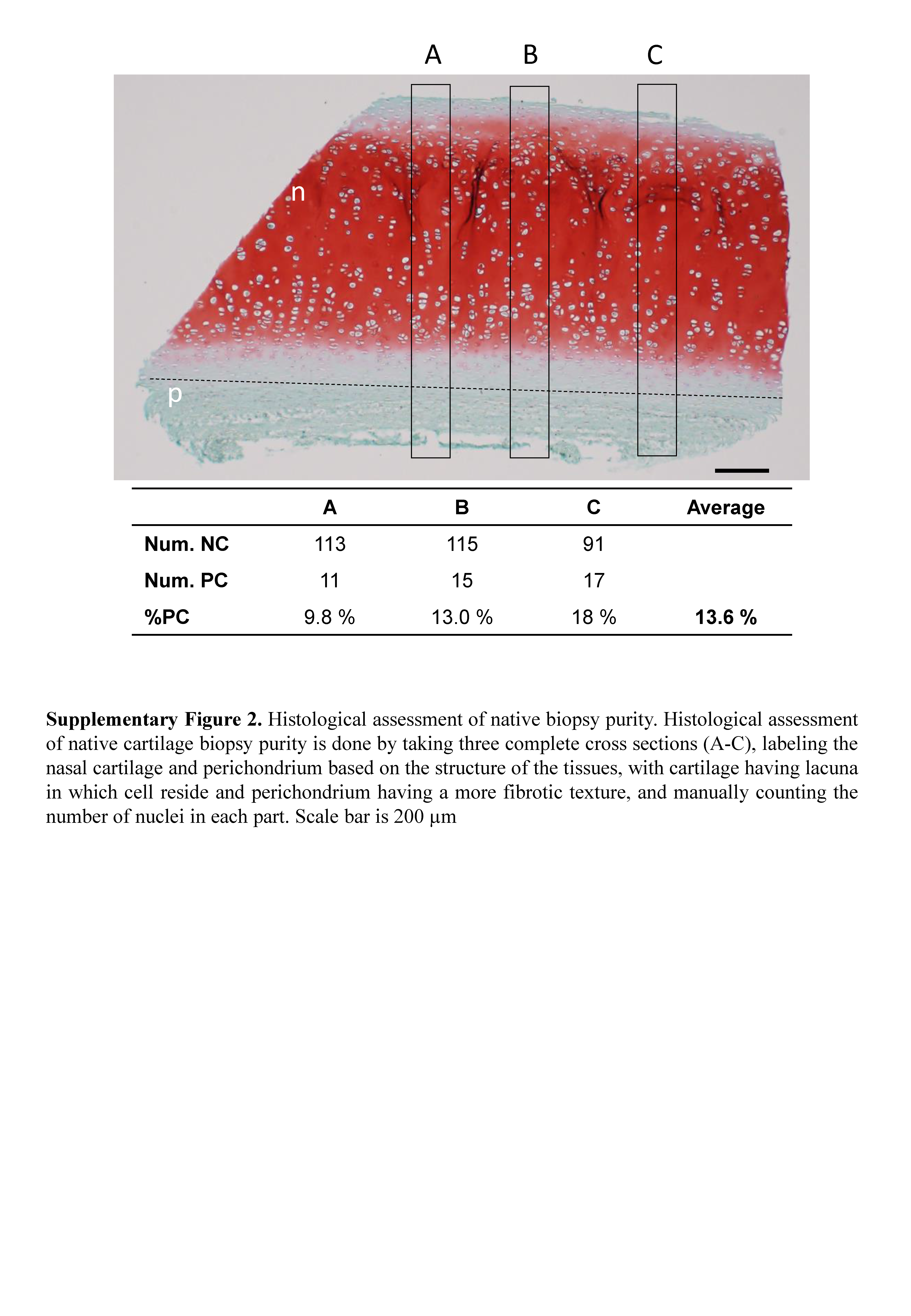

Supplement: Supplementary file 2 [file Image_2.TIFF]

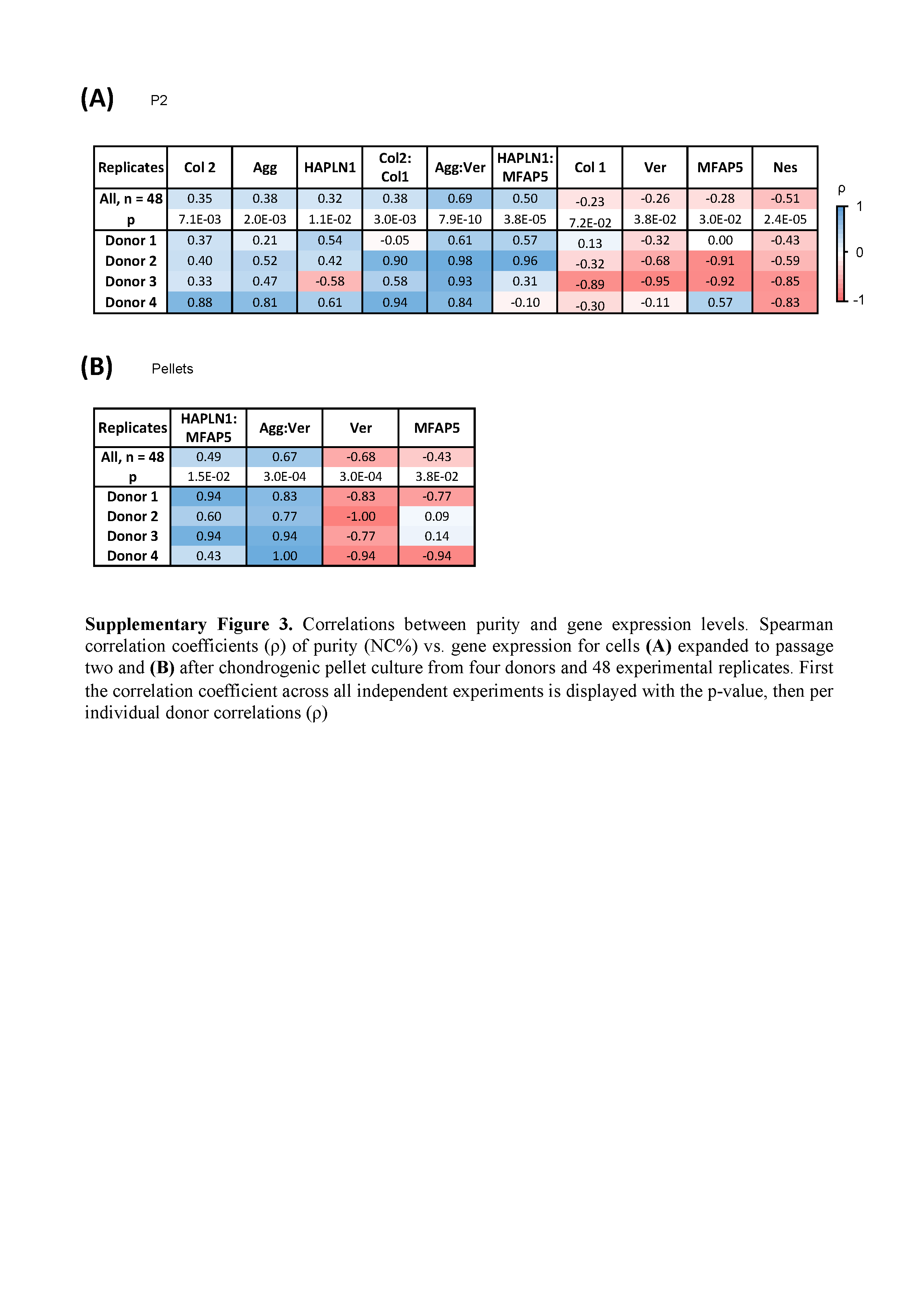

Supplement: Supplementary file 3 [file Image_3.tiff]

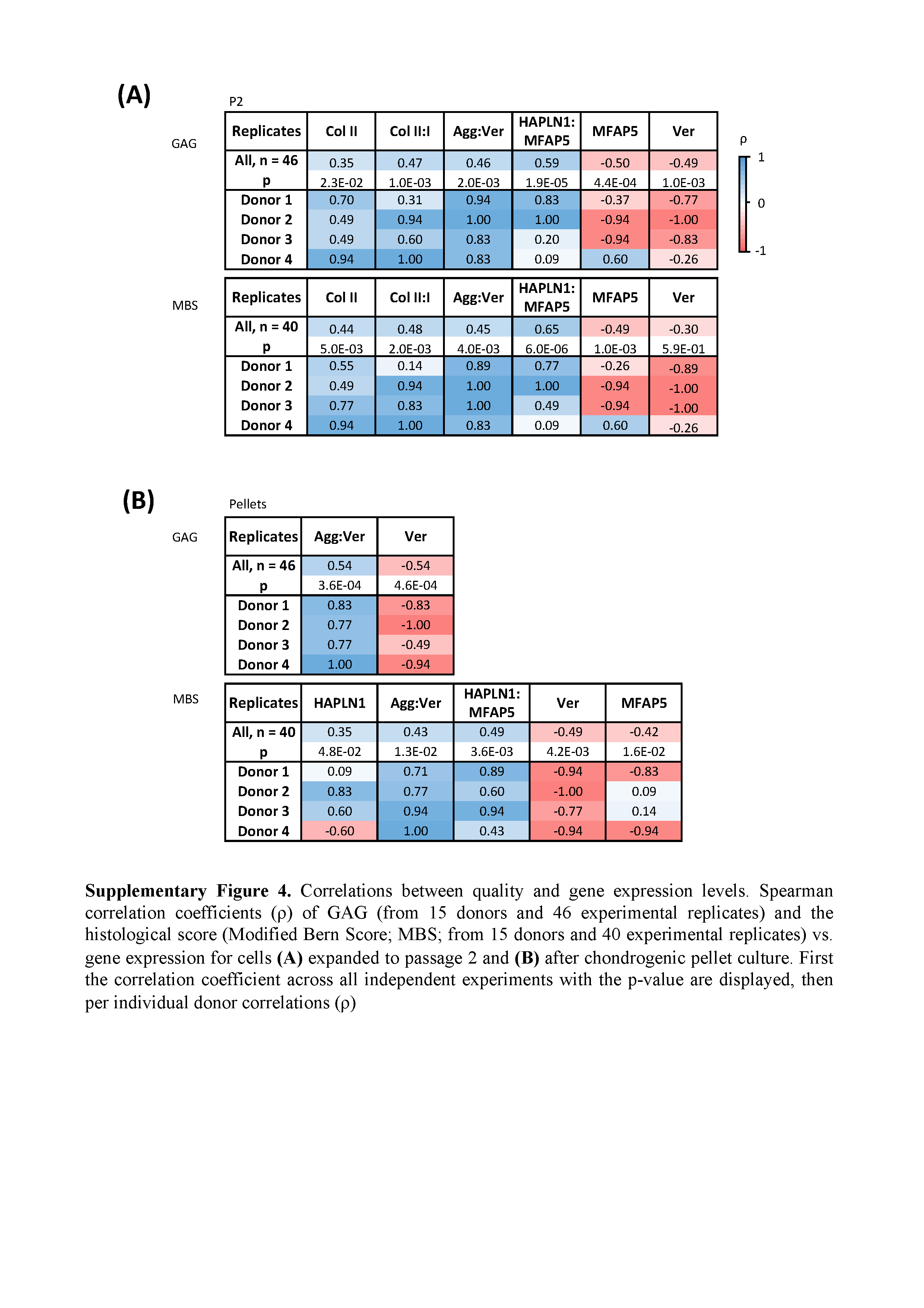

Supplement: Supplementary file 4 [file Image_4.tiff]

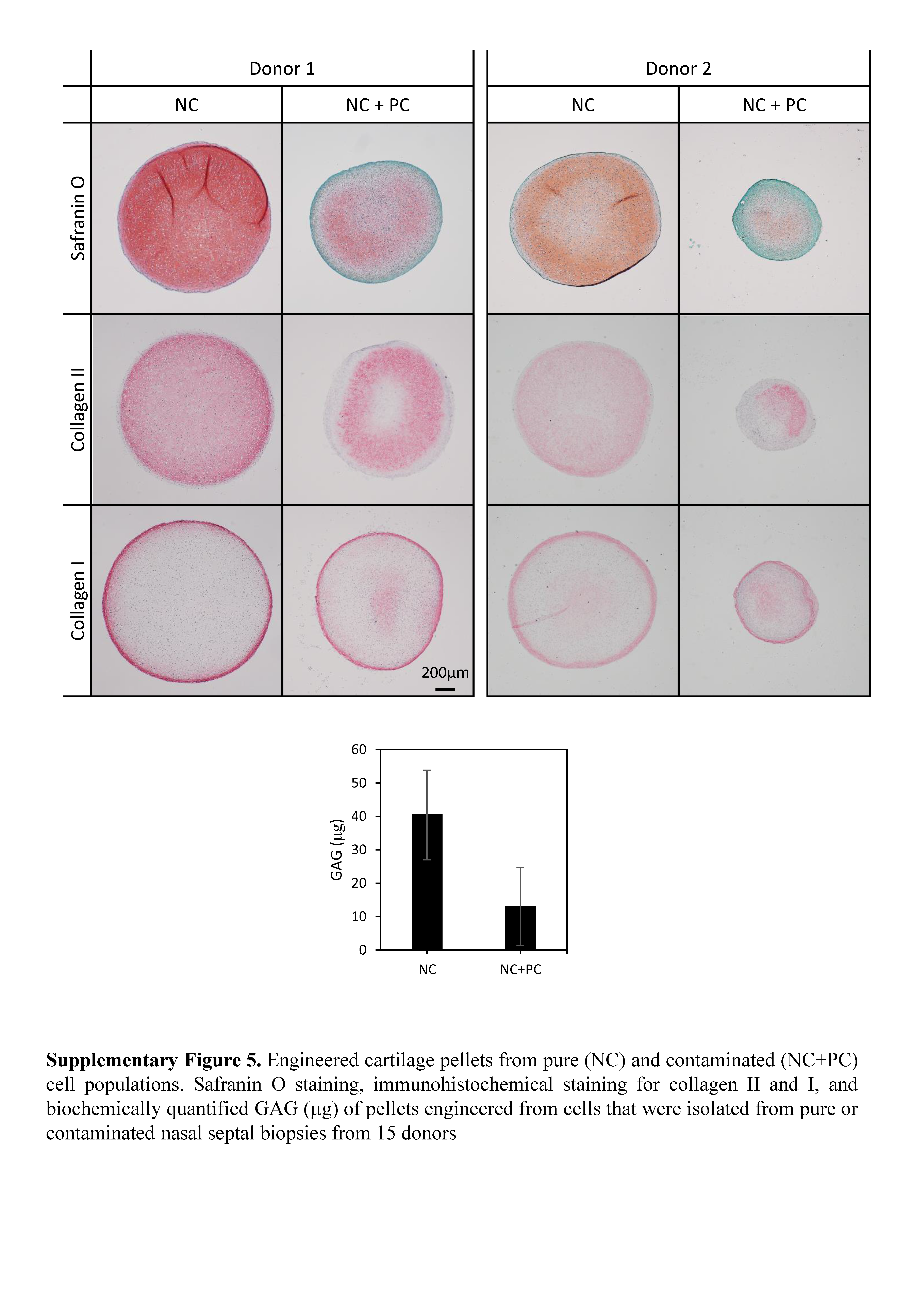

Supplement: Supplementary file 5 [file Image_5.TIFF]

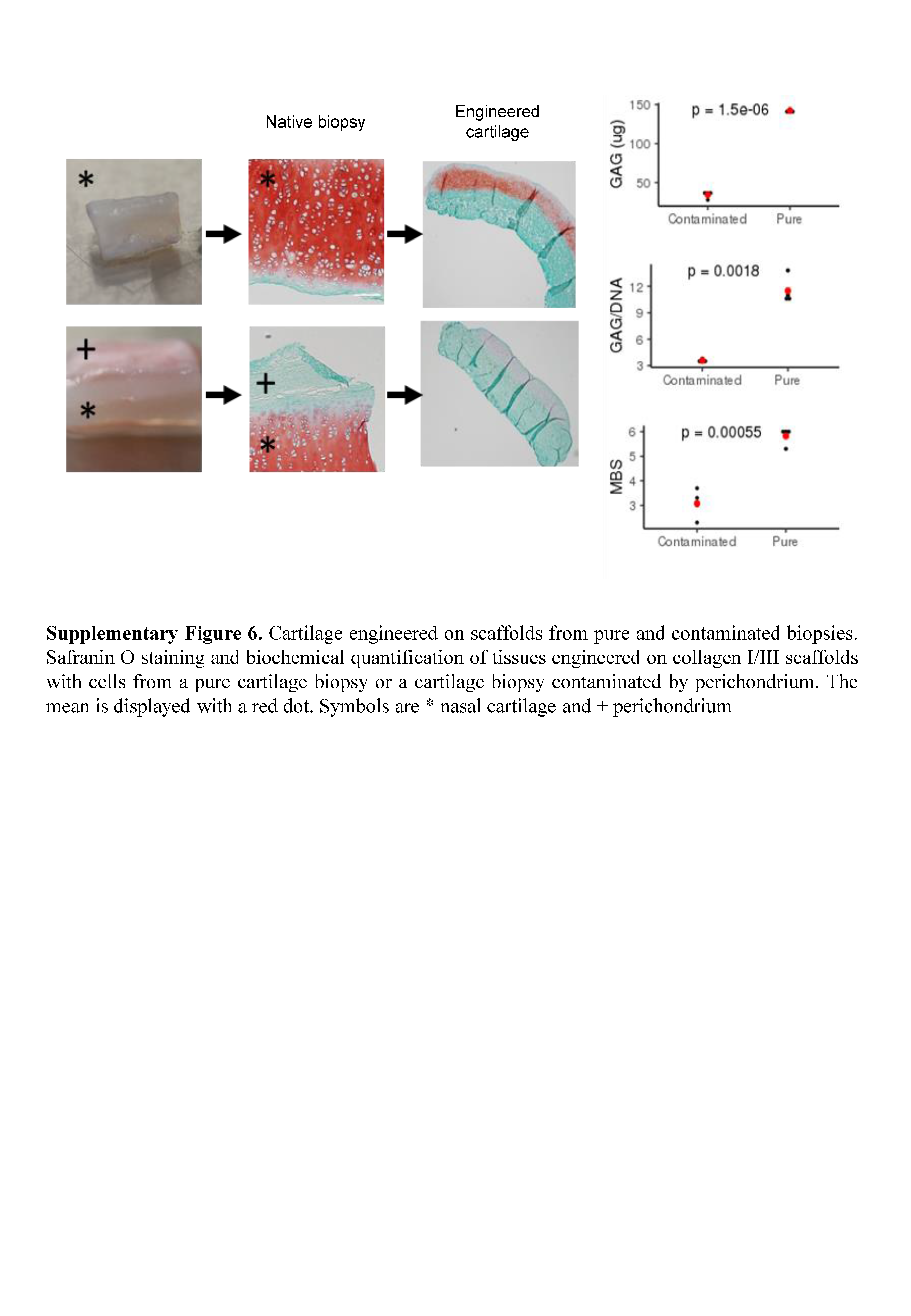

Supplement: Supplementary file 6 [file Image_6.TIFF]
